# Different pH requirements are associated with divergent inhibitory effects of chloroquine on human and avian influenza A viruses

**DOI:** 10.1186/1743-422X-4-39

**Published:** 2007-05-03

**Authors:** Livia Di Trani, Andrea Savarino, Laura Campitelli, Sandro Norelli, Simona Puzelli, Daniela D'Ostilio, Edoardo Vignolo, Isabella Donatelli, Antonio Cassone

**Affiliations:** 1 Dept. of Food and Animal Health, Istituto Superiore di Sanità, Rome, Italy; 2 Dept. of Infectious, Parasitic, and Immune-mediated Diseases, Istituto Superiore di Sanità, Rome, Italy

## Abstract

Chloroquine is a 4-aminoquinoline previously used in malaria therapy and now becoming an emerging investigational antiviral drug due to its broad spectrum of antiviral activities. To explore whether the low pH-dependency of influenza A viruses might affect the antiviral effects of chloroquine at clinically achievable concentrations, we tested the antiviral effects of this drug on selected human and avian viruses belonging to different subtypes and displaying different pH requirements. Results showed a correlation between the responses to chloroquine and NH_4_Cl, a lysosomotropic agent known to increase the pH of intracellular vesicles. Time-of-addition experiments showed that the inhibitory effect of chloroquine was maximal when the drug had been added at the time of infection and was lost after 2 h post-infection. This timing approximately corresponds to that of virus/cell fusion. Moreover, there was a clear correlation between the EC_50 _of chloroquine in vitro and the electrostatic potential of the HA subunit (HA2) mediating the virus/cell fusion process. Overall, the present study highlights the critical importance of a host cell factor such as intravesicular pH in determining the anti-influenza activity of chloroquine and other lysosomotropic agents.

## Background

A second look at selected compounds is giving new life to several abandoned therapies and new applications for existing drugs [1-3]. One such example is provided by chloroquine, being dismissed from antimalarial treatment and finding new applications in the clinical management of autoimmune diseases, tumours and non-malarial infections [4,5]. The use of chloroquine in the clinical management of a viral infection was first considered in the 1990s, on the basis of its effects on HIV-1 [6,7]. The drug is now being tested as an investigational antiretroviral [8].

Some of us previously analysed the reported effects of chloroquine on replication of several viruses and concluded that the drug should be studied as a broad spectrum antiviral agent against emerging viral infections, being relatively well tolerated, cheap, and immediately available worldwide [9]. As a weak base capable of accumulating within cellular organelles, chloroquine appears to be capable of interfering with pH-dependent steps in the replication of several viruses. Other mechanisms of viral inhibition by chloroquine, such as inhibition of polynucleotidyl transferases have, however, been considered [7]. In 2003–2005, chloroquine was studied as a promising *in vitro *anti-SARS agent [9-11] and recently entered clinical trials against chikungunya fever [12].

The broad-spectrum antiviral effects of chloroquine deserve particular attention in a time in which there are several cases of avian influenza A virus transmission to humans from poultry, and the availability of antiviral drugs is fundamental during preparation and evaluation of effective vaccines. Chloroquine inhibition of both type A and B influenza viruses was first described in the 1980s [13,14]. The concentrations employed in these studies were however too high to allow a theoretical transposition to *in-vivo *settings. Anecdotal reports of clinical benefits derived from a related compound, *i.e*. quinine, date back to the Spanish influenza pandemic of 1918/19. However, it was not until last year that the anti-influenza virus effects of chloroquine at clinically achievable concentrations were studied, in view of a possible application of this drug in the clinical management of influenza [4,15]. Investigations still have to be done on this topic. For example, the mechanisms of orthomyxovirus inhibition by chloroquine have been uncertain at the clinically achievable concentrations adopted in the most recent studies [4,15], as well as the effects of chloroquine on field isolates, including avian strains potentially transmittable to humans.

We here report the results of an initial evaluation of susceptibility to chloroquine of human and avian influenza A viruses. Susceptibility to chloroquine appears to be dependent on the pH requirements of the viruses and the electrostatic potential of haemagglutinin subunit 2 (HA2), which is involved in virus/cell fusion. Accordingly, the antiviral effects are exerted at an early step of virus replication.

## Results

**Figure 1:**
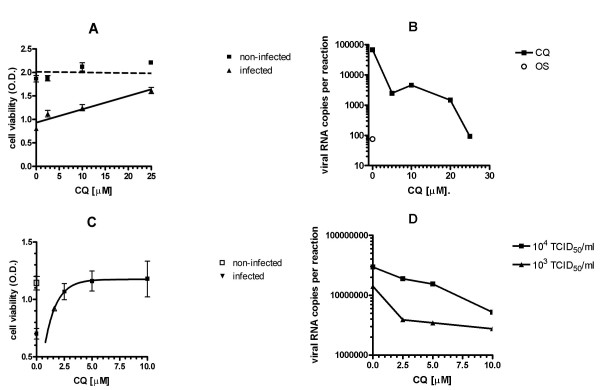
**Inhibition of H5 and H3 influenza A virus replication by CQ in MDCK cells**. Cells were incubated with chloroquine (CQ) after virus inoculation or mock-infection and tested for cell viability and viral RNA copies at 24 h post-infection. A) Viability of cells infected with A/Chicken/Italy/9097/97 (H5N9) and treated with increasing concentrations of CQ as detected by colorimetric test. Assays were performed as described in the text. The dotted line indicates inhibition of uninfected cell viability, the solid line indicates inhibition of infected cell viability. Results are presented as the curves that best fit the data points. B) Results of one representative experiment showing inhibition by CQ of A/Chicken/Italy/9097/97 viral RNA production. Virus infected MDCK cells were incubated for one day in the presence of 0, 5, 10, 20 or 25 μM chloroquine. Cell supernatants were used for viral RNA extraction and subjected to a quantitative real-time RT-PCR (qRRT-PCR) assay. Oseltamivir (OS; 20 nM) was used as a positive control. C and D) as in A and B), respectively, using A/Panama/2007/99-like (H3N2) virus. In D) both results obtained with inocula containing 10^4 ^and 10^3 ^TCID_50_/ml are reported. Results in B) and D) are displayed for purely representative reasons to show that there is inhibition of virus production, and cannot be compared with each other or with those in A) and C), due to the high intra- and inter-assay variability of the qRRT PCR assay (see Ref. [29]).

We first tested the effects of chloroquine on low-pathogenic (LP) A/Ck/It/9097/97 (H5N9) virus, isolated from poultry in Italy. We found that chloroquine dose-dependently inhibited the viral cytopathic effect with a 50% effective concentration (EC_50_) of 14.38 μM, in cells infected with the H5N9 virus at approx. 10^4 ^50% tissue culture infecting doses (TCID_50_)/ml (Fig. 1a). Although this value was rather high, some of the inhibitory concentrations matched the blood concentrations reported in individuals under acute antimalarial treatment (1–15 μM). The inhibitory effects were confirmed using quantitative reverse transcritptase real-time PCR (qRRT-PCR) (Fig. 1b).

Ooi *et al*. (2006) [15] recently reported that chloroquine inhibited human H3N2 and H1N1 viruses with EC_50 _values in the range of 0.84 – 3.60 μM. To investigate whether the discrepancies with the inhibitory values reported above were due to the type of virus or to the different conditions and methods adopted, we tested the effects of chloroquine on replication of recent human H3N2 and H1N1 viral isolates under conditions similar to those adopted for the H5N9 avian influenza virus. Using the test of inhibition of viral cytopathogenicity, we found that chloroquine inhibited the H3N2 virus (10 ^4 ^TCID_50_/ml) with an EC_50 _of 1.53 μM (Fig.1c). Inhibition was confirmed using qRRT-PCR, both under similar conditions and at lower MOIs (Fig.1d), thus confirming the results of Ooi *et al*. (the assay adopted by these authors employs a lower MOI than routinely used by our group). Results obtained with H1N1 viruses (10^4 ^TCID_50_/ml) showed a similar drug susceptibility for the human strain (EC_50 _= 1.26 μM), in full agreement with Ooi *et al*. [15], but no response to clinically achievable drug concentrations in an avian strain (IC_50 _> 20 μM; data not shown). These data suggest a more pronounced inhibitory effect of chloroquine on human H3N2 and H1N1 viruses than on avian H5N9 virus replication.

Since 1) chloroquine is thought to interfere with pH-dependent steps of the life cycles of several viruses [9], and 2) some of us reported different pH requirements in influenza A viruses infecting different avian species [15], we investigated whether response of influenza viruses to chloroquine might depend on the different pH requirements of the human and avian viruses.

Thus, we analysed the response of human H3N2 virus (good chloroquine responder) and avian H5N9 virus (poor chloroquine responder) to ammonium chloride (NH_4_Cl; 40 mM), a lysosomotropic agent known to increase the pH of intracellular vesicles. Results showed a good response of the chloroquine-sensitive H3N2 virus to NH_4_Cl inhibition of viral cytopathogenicity (100% inhibition under conditions described above). Conversely, the lower chloroquine-sensitivity of H5N9 virus was associated with lack of response to NH_4_Cl (data not shown). This observation raised the hypothesis that cellular pH might be a critical factor for chloroquine inhibition of influenza virus.

To explore this possibility, the action of chloroquine was tested on two avian H7N3 viruses whose haemagglutinins (HAs) differ in two amino acid positions (*i.e*. residue 261 in the HA1 subunit and residue 161 in HA2, the latter being the HA subunit mediating the fusion process), and which display different pH requirements [16]. The two viral strains showed a marked discrepancy in the response to chloroquine. In particular, A/Mallard/It/43/01 (H7N3) virus, which had been shown to be relatively more independent from pH increase than A/Ty/It/220158/02 virus [16], was insensitive to clinically achievable chloroquine concentrations (EC_50 _> 20 μM). In contrast, chloroquine exerted some inhibitory effect on A/Ty/It/220158/02 replication (EC_50 _= 14.39 μM), although the response of this virus to chloroquine was lower than that of H3N2 virus. The different pH requirements of the two viruses were confirmed by the different responses to NH_4_Cl (0% inhibition for A/Mallard/It/43/01; 30% inhibition for A/Ty/It/220158/02). This result raises the hypothesis that chloroquine inhibits pH-dependent events involving HA.

**Figure 2:**
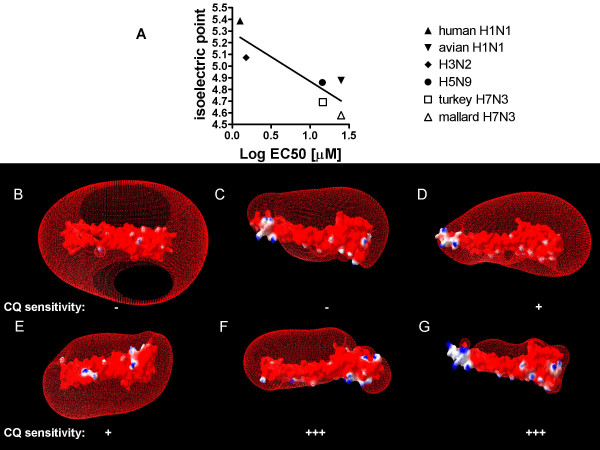
**Correlation between electric characteristics of haemagglutinin subunit 2 (HA2) and response to chloroquine of influenza A viruses. **A) Correlation between EC_50 _of chloroquine (CQ) on viral cytopathogenicity (presented as *Log *values, *x *axis) and isoelectric point of HA2 (pH value at which the protein is neutral; *y *axis). The line best fitting the data points is shown. Isoelectric points were calculated based on the protein sequence using the web interface in Ref. [35]. B-G) Theoretical three-dimensional models for HA2 subunits of the viruses adopted in the present study, shown in ranked order of sensitivity to chloroquine (from resistant to clinically achievable concentrations to fully sensitive). B) A/Mallard/Italy/43/01 (H7N3); C); A/Mallard/Italy/66/96 (H1N1); D) A/Chicken/Italy/9097/97 (H5N9); E) A/Turkey/Italy/220158/02 (H7N3); F) Human influenza A/Panama/2007/99-like (H3N2); G) A/New Caledonia/20/99-like (H1N1). The electrostatic potential is mapped to the protein surface as a range of colours from red (-1.8 V) to blue (+1.8 V). Equipotential surfaces are shown as dotted line grids. Protein sequence data regarding the H7N3 and H5N9 viruses can be found in GenBank with the following accession numbers: AAT37405, AAT37404, AAG60349.

To further explore this possibility, the isoelectric point was calculated for HAs of all viruses used in the present study, and the electrostatic potential was mapped on the protein surfaces of 3D models obtained by homology with the crystal structures of matched subtype representatives. Results showed a negative correlation between the isoelectric point of HA2 and the IC_50 _of chloroquine *in vitro *(*r *= -0.87; *P *= 0.024; Fig. 2). Instead, no correlation was found with the isoelectric point of HA1 (*P *> 0.05; data not shown). Although the viruses studied here belonged to different subtypes, chloroquine-resistant viruses, independently of the subtype, showed a more marked negative surface potential of HA2 than chloroquine-sensitive viruses (Fig. 2). Viruses with intermediate drug sensitivity showed intermediate characteristics (Fig. 2). We conclude that structural determinants in HA2 are associated with response of influenza A viruses to chloroquine.

If the hypothesis of a pH dependent inhibitory action of chloroquine was correct, the timing of drug inhibition should match that of virus/cell fusion, an early step of virus replication occurring in endosomes and requiring a low pH (approx. pH 5–5.5) in several, but not all, influenza A viruses, as shown by previous studies [17]. As the assays for detection of antiviral effects adopted in the first part of this study were designed to allow multiple cycles of viral replication, we designed time-of-addition experiments using the chloroquine-sensitive human H3N2 virus. Chloroquine was added during virus adsorption onto cells (*i.e*. time 0; T_0_) and/or at 1, 2, 3 and 4 h post-infection (T_1–4_) Using qRRT-PCR, we found that the inhibitory effect of chloroquine was highest when the drug was added at T_0 _(inhibition of viral replication corresponding to 89,36%) and at T_1 _(inhibition of replication corresponding to 15,53%), whereas the inhibitory activity was completely lost at T_2_.

If this timing was correct, chloroquine should inhibit influenza A replication by a novel mechanism, and therefore exert additive effects in combination with oseltamivir, inhibiting neuraminidase activity at the late stages of viral replication cycle. To test this hypothesis, human H3N2 virus-infected cells were treated with different chloroquine concentrations in the presence or absence of oseltamivir (10 nM). The virus-infected cells were also incubated with oseltamivir alone, EC_50_= 20 nM. Isobologram analysis showed that the two drugs exerted an additive effect (sum of FICs = 1) (data not shown). This result provides further evidence that chloroquine inhibits viral replication by a mechanism different from that of one major anti-influenza drug.

## Discussion

The results so far obtained suggest that chloroquine inhibits the replication of those influenza A viruses requiring low pH for proper fusion activation and that the antiviral effects occur at an early stage of viral replication. Supporting evidence comes from: 1) the more sustained inhibitory effect of chloroquine on those viruses whose haemagglutinins (HAs) were found to require low pH for their fusion activity, 2) common HA2 characteristics such as surface potential and isoelectric point in chloroquine-sensitive viruses, and 3) time-of-addition experiments with H3N2 virus.

Chloroquine was found to inhibit a number of cellular processes, some of which do not depend on low pH but might anyway interfere with viral replication. For example, the drug was found to inhibit viral nucleotidyl transferases such as HIV-1 integrase [7]. If chloroquine inhibited influenza A RNA-dependent RNA polymerase, the timing of viral inhibition would not be consistent with that observed in the present study, because RNA replication occurs in the nucleus at later stages [18].

Based on bioinformatic studies, it was recently hypothesized that chloroquine might inhibit UDP- N acetylglucosamine transferase [1], a limiting enzyme in sialic acid synthesis. This specific issue has not been addressed here. Nonetheless, if the antiviral effect of chloroquine reported here were due to inhibition of UDP-N acetylglucosamine transferase, the drug should likely have antagonized the antiviral effect of the neuraminidase inhibitor oseltamivir (acting on detachment of sialic acid-bound virions from parent cells), rendering oseltamivir inhibition unnecessary. Instead, chloroquine was found in the present study to exert antiviral effects that were additive to those of oseltamivir.

Chloroquine is a weak base that is known to affect acid vesicles leading to dysfunction of several proteins [9], and has been shown to inhibit different viruses requiring a pH-dependent step for entry [19-22]. The results of the time-of-addition experiments, performed in the present study using a recent epidemic isolate of human H3N2 virus, are consistent with chloroquine inhibition of pH-dependent steps occurring at an early phase of influenza A virus replication.

Most influenza viruses enter target cells by fusion of the viral and cell membranes at the endosomal pH (approx. pH 5–5.5), although some virus variants can replicate well also at higher pH values [16,17,23]. In a previous paper, the differential growth sensitivity of two naturally occurring variants of an H7N3 virus, isolated from mallards and turkeys, to increased pH values was shown to correlate with different fusion properties [16]. Since chloroquine appears to mimic the effects observed on these two viruses when pH is increased, our data support the hypothesis that the step of virus replication inhibited by clinically relevant chloroquine concentrations is the low-pH dependent haemagglutinin-mediated virus/cell fusion, in agreement with the evidence obtained by other authors at much higher concentrations than those used in this study [13,14]. The correspondence between antiviral effects and isoelectric point of HA2 (*i.e*. the HA subunit mediating the fusion process) is also consistent with this mechanism. Acidic pH in the endosomal compartment also activates the influenza virus ion channel, M2, that promotes the uncoating of influenza virus in endosomes. However, M2 involvement as a possible target of the antiviral effect of chloroquine is unlikely, since no aminoacid differences were observed in M2 trans-membrane region (the ion-channel domain) between the two avian H7N3 viruses with different chloroquine sensitivities, as already reported [16].

Although a comprehensive study on the variation of fusion pH requirements of influenza A viruses of all HA subtypes and isolates from different hosts is not available, several authors have documented that the threshold pH, at which the HA conformational change and virus-cell fusion occur, is strain-specific [24-26]. Interestingly, the viruses showing highest chloroquine sensitivity also displayed the highest HA2 isoelectric points. Thus, a relationship between isoelectric point and response to pH is apparent. However, a broad study relating the surface electrostatic potential with inactivation by pH would be required to analyse the molecular details of the HA/pH interplay. Analyses of virus production before and after exposure to chloroquine and of the possible changes in HA2 surface potential in viruses rendered resistant to chloroquine after long exposure to the drug will also be necessary.

## Conclusion

Although association between variables cannot be considered to be equivalent to causation, the results of the present study strongly suggest that pH critically determines the antiviral activity of chloroquine by regulating virus/host cell interactions. The potential use of this compound as an antiinfluenza drug should take into consideration the possibility that even within the same subtype, different strains may present significantly divergent sensitivities to chloroquine as a consequence of their different pH requirements. Moreover, sensitivity to chloroquine may vary in different cell populations susceptible to influenza A virus infection, depending on different capabilities of endosome acidification. Mutations affecting the electrostatic potential of the the HA2 protein subunit of various isolates of the same virus could also be relevant. All these factors should be carefully evaluated when hypothesising a potential clinical utilisation of chloroquine against influenza A viruses.

## Materials and methods

### Cells and virus stocks

Madin Darby Canine Kidney (MDCK) cells were obtained from the American Type Culture Collection. The following viruses were used in this study: two recent human strains, A/Panama/2007/99-like (H3N2) and A/New Caledonia/20/99-like (H1N1), and four LP avian influenza viruses, A/Chicken/Italy/9097/97 (H5N9), A/Turkey/Italy/220158/02 (H7N3) and A/Mallard/Italy/43/01 (H7N3), A/Mallard/Italy/66/96 (H1N1). Virus titration was performed by 50% tissue culture infectious dose (TCID_50_) in MDCK cells, as described [27], and virus stocks were aliquoted and stored at -70°C until used. All the viruses were from the Istituto Superiore di Sanità (ISS) repository. Virus infection of MDCK cell monolayers was carried out according to standard procedures [28].

### Compounds

Chloroquine phosphate (7-chloro-4- [4-(diethylamino)-1-methylbutyl]amino]quinoline phosphate, (Sigma) and oseltamivir, a kind gift from Roche was used as a positive control.

### Virus yield assay

After 24 hours of incubation of the virus-infected cells with different concentrations of the test compounds, under the appropriate conditions, pooled aliquots of the supernatants containing free viruses were subjected to RNA extraction and qRRT-PCR.

### Quantitative Real Time RT-PCR (qRRT-PCR)

A one-step qRRT-PCR assay was employed, which makes use of minor groove binder (MGB) probe technology, as previously described [29]. Briefly, viral RNA was extracted from infected cell supernatants (QIAmp Viral RNA Mini kit – Qiagen, GmbH, Hilden, Germany) and amplified by RRT-PCR using primers and probe targeting a highly conserved region of the matrix gene of influenza type A viruses. The influenza matrix RNA was also *in vitro *transcribed from the corresponding DNA template, cloned into a plasmid vector as previously described [29] and used as standard RNA to generate standard curves for quantification of the vRNA in cell supernatants.

### Assay for measurement of antiviral activity

100 μl of 1.5 × 10^5 ^cells/ml MDCK cells in growth medium was seeded into each well in the 96-well microtiter plate. When the cell monolayer was confluent, the culture medium was removed and cells were washed twice with serum-free MEM. Then, 100 μl of the Type A influenza viruses under study containing 10^3 ^– 10^4 ^TCID_50 _were inoculated in wells and the plates incubated for 1 hour at 37°C in humidified air of 5% CO_2_. The viral suspension was removed and the cells were washed two times; fresh medium containing TPCK-trypsin and chloroquine at different concentrations or NH_4_Cl (40 mM) was then added to culture wells in triplicate. Antiviral activity and cytotoxicity measurements were based on the viability of cells that had been infected or mock infected with influenza viruses in the presence of various concentrations of the test compounds. One to three days, depending on the kinetics of cytopathogenicity, after infection the number of viable cells was quantified by a tetrazolium-salt-based-colorimetric method (CellTiter 96 AQueous One Solution kit, Promega, The Netherlands).

### Assessment of the effects of two drugs in combination

To measure the anti-influenza effects of chloroquine/oseltamivir drug combinations, cell pellets were resuspended in media containing increasing concentrations of the antimalarial in the presence or absence of oseltamivir. A fractional inhibitory concentration (FIC) was then calculated as the ratio: 50% effective concentration (EC_50_) of drug A in combination with drug B/EC_50 _of drug A alone. The effect was considered to be additive when the sum of FICs was between 0.8 and 1.2, as previously described [8]

### Time-of-addition assay

Monolayers of MDCK cells in 96-well plates were infected with 100 μl of medium containing approximately 10^4 ^TCID_50 _of H3N2 subtype. After 1 hour of adsorption, cell monolayers were washed twice with serum-free MEM and incubated in fresh medium containing TPCK-trypsin and chloroquine at a concentration of 10 μM. Chloroquine was added at the time of infection or at four different time points thereafter. Eight hours post-infection, a time point at which all progeny virus in the supernatants is derived from the first replication cycle, cell supernatants were collected, viral RNA was extracted and the antiviral activity was determined by using the qRRT-PCR described above.

### Viral RNA sequencing

Hemagglutinin genes of H3N2 and H1N1 viruses were sequenced using gene-specific primers, as previously described [30]. Sequence data so far unpublished will be deposited in GenBank by the time of publication of the present article.

### Molecular modelling

Three-dimensional models for the HAs of the viruses used in the present study were obtained by homology modelling, using the SWISS model web server [31,32], using, as templates, structures of matched subtype representatives deposited in the protein data bank (PDB) [33]. Hydrogens were added using VEGA-ZZ (University of Milan, Italy) [34], and the structures were then visualised using the Swiss PDB Viewer (SPDBV) program (Swiss Institute of Bioinformatics) [31].

The Coulomb potential was mapped to the protein surface by use of SPDBV using the default relative dielectric constant (solvent = water) of 80. Further information on the algorithm adopted by the program is available in the detailed online description of the program [31].

## Authors' contributions

LDT developed the virus detection assays and participated in the experimental design and manuscript drafting. AS participated in the general design of the study, data analysis and molecular modelling, and drafted the manuscript. LC participated in the experimental design, data analysis and manuscript drafting. SP prepared and sequenced the viral samples and was involved in the assays for antiviral activity. EV, SN and DDO conducted the assays for antiviral activity, ID coordinated the virological activities and participated in the experimental design. AC conceived and coordinated the entire study.
